# Analysis of Relations Between the Level of Mg, Zn, Ca, Cu, and Fe and Depressiveness in Postmenopausal Women

**DOI:** 10.1007/s12011-016-0798-9

**Published:** 2016-07-30

**Authors:** Małgorzata Szkup, Anna Jurczak, Aleksandra Brodowska, Agnieszka Brodowska, Iwona Noceń, Dariusz Chlubek, Maria Laszczyńska, Beata Karakiewicz, Elżbieta Grochans

**Affiliations:** 1 Department of Nursing, Pomeranian Medical University in Szczecin, Żołnierska 48, 71-210 Szczecin, Poland; 2 Clinic of Gynecology and Urogynecology, Pomeranian Medical University in Szczecin, Siedlecka 2, 72-010 Police, Poland; 3 Department of Medical Chemistry, Pomeranian Medical University in Szczecin, Powstańców Wlkp. 72, 70-111 Szczecin, Poland; 4 Department of Biochemistry and Medical Chemistry, Pomeranian Medical University in Szczecin, Powstańców Wlkp. 72, 70-111 Szczecin, Poland; 5 Department of Histology and Developmental Biology, Pomeranian Medical University in Szczecin, Żołnierska 48, 71-210 Szczecin, Poland; 6 Department of Public Health, Pomeranian Medical University in Szczecin, Żołnierska 48, 71-210 Szczecin, Poland

**Keywords:** Depressiveness, Postmenopause, Bio-elements, Cu, Mg

## Abstract

Numerous observations suggest a possible connection between the levels of Mg, Zn, Fe, and Zn and the incidence of depressive symptoms. Depression is two to three times more common in women than in men. The menopausal period is extremely conducive to depressive disorders. The aim of this study was to assess the severity of depressive symptoms in postmenopausal women depending on the levels of Mg, Zn, Ca, Cu, and Fe. The study included 198 healthy postmenopausal women at the average age of 56.26 ± 5.55 years. In the first part of the study, standardized research tools were used, namely the Primary Care Evaluation of Mental Disorders (PRIME-MD) and the Beck Depression Inventory (BDI). The second part involved biochemical analysis of Mg, Zn, Ca, Cu, and Fe levels in blood serum. The lowest Cu levels were observed in women without depressive symptoms (1.07 ± 0.22 mg/l) and the highest in those with severe depressive symptoms (1.19 ± 0.17 mg/l), (*p* ≤ 0.05). The lowest Mg levels were observed in women with depressive symptoms (14.28 ± 2.13 mg/l), and the highest in women without depressive symptoms (16.30 ± 3.51 mg/l), (*p* ≤ 0.05). The average serum Mg levels (15.75 ± 3.23 mg/l) decreased compared to the reference values (18.77–24 mg/l). What is striking is a potential relation between the levels of Mg and Cu and depressiveness. Our results indicate to a higher vulnerability to depression in a group of women with lower levels of Mg and higher levels of Cu.

## Introduction

Depression is a mental disorder that is characterized by high morbidity and mortality. According to estimates of the World Health Organization, depression is the fourth major cause of disability among people that depends on years of life. It is expected that by the year 2020 depression will have occupied the second place in the classification for several years [1]. Numerous observations suggest a possible connection of the level of Mg, Zn, Fe, and Zn and the frequency of occurrence of depressive symptoms [2].

Depression is a disorder that predominates females (women suffer two to three times more often than men) [3]. The menopausal period is extremely conducive to the occurrence of depressive disorders [4]. This often involves hormonal changes that result in the end of the reproduction period, cessation of menstruation, vaginal dryness that leads to reduced libido, dyspareunia, frequent infections in the urogenital system, urinary incontinence, and pelvic organ prolapse. In the perimenopausal period, 20–30 % of women experience depressive disorders that require treatment, and up to 90 % of women show weaker mood disorders i.e. difficulty concentrating, irritability, and emotional lability as a direct result of hormonal changes [5].

Menopause usually occurs between 47 and 51 years of age, and the period of life of women after the last menstrual period, regardless of whether menopause was natural or artificial, is defined as postmenopause [6]. In Poland, menopause falls on average on 43–55 years of age, and the average age of experiencing menopause is 50.8 years [7]. In accordance with the latest classification proposed by the 2001 Stages of Reproductive Aging Workshop (STRAW), the postmenopausal period is characterized by an increase in the FSH level and a decrease in the oestradiol level. Changes in the levels of these hormones are particularly visible in the first two years after the last menstrual period, and then the hormone levels stabilize. The postmenopausal period begins after 12 months without menstruation [8].

The average lifespan for women has considerably increased since the beginning of the last century, causing that currently approximately 30 % of women’s lives is spent in the postmenopausal period. Therefore, women’s health problems associated with menopause and ovarian steroid deficiency have become an important issue for public health. More and more often researchers focus on looking for biological factors that affect depressiveness as in this case micronutrients may be important.

Mg is a bivalent intracellular cation which acts as a coenzyme or an activator for approximately 300 enzymatic systems, especially for enzymes involved in the transfer of phosphate groups and energy changes in a cell [9]. The regulating effect of Mg exerted on N-methyl-D-aspartate (NMDA) channels makes it an important factor in the treatment of depression [10]. Prolonged Mg deficiency can lead to the development of atherosclerosis, cardiovascular diseases, and metabolic disorders. Mg deficiency can cause endocrine disorders i.e. hyperthyroidism, primary aldosteronism, hyperparathyroidism, and neurological disorders such as paraesthesia, increased muscle contraction, convulsions, and loss of consciousness [11]; it also promotes development of mental disorders [12]. Hypomagnesaemia occurs in situations of chronic stress, alcohol abuse, diet rich in carbohydrates and fats, or containing increased amounts of Ca, as well as situations of increased physical activity and elimination diets (especially when combined with the use of laxatives) [13].

Zn is an essential micronutrient necessary for normal cellular metabolism in the human body. It plays a fundamental role in a wide range of biochemical processes. Zn is an important modulator of functioning of the central nervous system [14]. Zn deficiency in humans is relatively rare. It was described in cases of emotional stress, and such diseases as giardiasis, diarrhoea, acute pancreatitis, and chronic renal failure. Prolonged Zn deficiency is manifested, among others, in neuropsychiatric disorders such as apathy, depression, and lack of concentration [15].

Zn and Mg deficiency in women can significantly reduce their quality of life, causing palpitations, trembling hands and feet, paraesthesia, impaired immune system, symptoms of dryness and roughness of the skin, hair loss, apathy, depression, impaired concentration, and impaired vision, hearing, taste, and smell [9].

Ca is a bivalent cation that is predominantly accumulated in bone tissue (i.e. in 99 %). Some authors believe that the main cause of instability of the atherosclerotic plaque is the deposition of Ca deposits in place of inflammation [16]. Hypocalcaemia manifests itself in fragility of hair and nails, and it can also lead to mood disorders, irritability, depression, and increased sleepiness. In the course of hypocalcaemia, there may be dizziness and even loss of consciousness [17].

Another microelement important for the functioning of the human body is Cu, which mainly accumulates in the liver, muscles, the skeletal system, and the brain. If the concentration of Cu in blood serum is too high it results in damage to the kidneys, liver, and coronary arteries. Cu deficiency can manifest itself in disorders of the circulatory system, the nervous system, the digestive system, and it can cause loss of hair pigmentation [18].

Fe is mainly a component of haemoglobin, myoglobin, and some enzymes (cytochromes, catalase, peroxidase). It is delivered with food and absorbed in the small intestine; later, it is excreted in faeces with scaly cells [19]. In the human body, it transports oxygen through haemoglobin. The most common cause of Fe deficiency is poor diet, malabsorption, blood loss, and chronic inflammation. Due to limiting of aerobic changes in muscles and advantage of anaerobic changes due to the low concentration of Fe, its deficiency is manifested mainly in weakness, and a decrease in the overall efficiency of the human body. There are also skin lesions in the form of tongue inflammation, lip sores in the corners of the mouth, itching, fragility, and brittle hair and nails. Moreover, a decrease in body temperature and appetite can be observed. Some authors associate the restless leg syndrome with Fe deficiency [20, 21].

Excess of Fe is due to excessive storage of this micronutrient in the course of hemochromatosis, also known as brown diabetes. This leads to multiple organ complications. Fe accumulates in the liver, pancreas, myocardium, and the skin mainly in the form of haemosiderin [22].

The aim of the study was to:

1. Assess the concentration of Mg, Zn, Ca, Cu, and Fe in blood serum in postmenopausal women.
2. Assess the severity of depressiveness depending on the concentration of Mg, Zn, Ca, Cu, and Fe in postmenopausal women.

## Material and Methods

The study included 198 healthy postmenopausal women, residents of the West Pomeranian Province in Poland. Women were chosen according to the following criteria: postmenopausal period confirmed by absence of menstruation for at least a year, no menopausal hormone therapy (MHT) in medical history, no current psychiatric treatment, no tumours, lack of thyroid diseases and diabetes, lack of addictions (alcohol and cigarettes), correct blood pressure parameters, no dietary supplements and any drugs.

The research was approved by the [hidden for blind review], No. KB-0012/154/12.

The first part of the research was carried out using the diagnostic survey method. The following standardized research tools were used:

- PRIME-MD questionnaire to exclude women with axis I mental disorders according to ICD-10 [20].
- Beck’s depression scale—general physical and mental state self-study for determining the severity of depressiveness. The following standards were used: no depression (0–11), mild depression (12–26), moderate depression (27–49), and heavy depression (50–63).

The study sample was divided according to the severity of depression (no depression, mild, moderate, and heavy depression) and according to the presence or absence of depression.

The second part of the research included biochemical studies of the concentration of bio-elements: Mg, Zn, Ca, Cu, and Fe in blood serum.

After obtaining consent to participate in the study from each patient, 5 ml of blood was taken to determine the concentration of the following microelements in serum: Mg, Ca, Cu, Fe, and Zn. Blood samples were collected with the Vacutainer on an empty stomach (at least 8 h after the last meal). Biological material was then centrifuged (10 min, 4000 rpm/min) and storage in –20 °C. After being thawed, blood serum was mixed and diluted with distilled water. Serum samples were diluted 1:2 to determine the concentrations of Zn, Cu, and Fe and 30:3 to determine the concentrations of Ca and Mg. Zn, Cu and Fe, Ca and Mg were determined using the atomic absorption spectrometry and an atomic absorption spectrometer—PU 9100X by Philips. Concentrations were read using the designated patterns of the Titrisol® curve by Merck. Working wavelength for Zn—213.9 nm, for Fe—248.3 nm, and for Cu—354.8 nm, for Ca—422.7 nm, and for Mg—285.2 nm. When determining Ca and Mg, the lanthanum chloride solution was used as the ionization buffer. Determinations were made using the acetylene-oxygen flame.

Results of the control studies were assessed twice according to the adopted 2SD rule by Westgard, which says that if in the control study the result of reproducibility is outside the 2SD range, it is necessary to make the determination in the control sample one more time. If the result is within the range of expected values it is assumed that the deviation in the first study was random in nature, and the results of the whole series are correct. However, if the second analysis demonstrates a value that is beyond the 2SD range once again, it must be assumed that the whole series of determinations associated with this sample is also encumbered with an error, so the results cannot be considered reliable (the series rejection principle). In such cases, the cause of an error was identified, and then the control and the series of determinations were repeated.

The results of the control were documented and written in Levey-Jenings’ control charts. Laboratory tests were carried out in the Department of Chemistry and Biochemistry at the Pomeranian Medical University in Szczecin.

The standard laboratory reference was the concentration range of Mg—18.77–24 mg/l, Fe—0.5–1.7 mg/l, Ca—81–105 mg/l, Zn—0.75–1.3 mg/l, and Cu—0.97–1.17 mg/l.

Statistical analysis was performed using Statistica PL. The study sample was characterized by quantitative variables, using minimum and maximum values, arithmetic mean, and standard deviation. The differences were assessed by means of the Mann-Whitney *U* test and the Student’s *t* test. Correlations between two variables were analysed using Spearman’s rank correlation coefficient. The level of significance was set at *p* ≤ 0.05.

## Characteristics of the Study Group

The average age of the studied women was 56.26 ± 5.55, the median was 55 years, the youngest respondent was 42 years old, and the oldest was 70 years old. More than half of the studied women had secondary education—56 % and higher education—32 %. Most women were residents of cities, including 62 % of cities over 100,000 residents, and 23 % of smaller towns with population of 10,000–100.000 people. Married women accounted for 69 % of the respondents, professionally active women—65 % of the respondents.

## Results

**Fig. 1:**
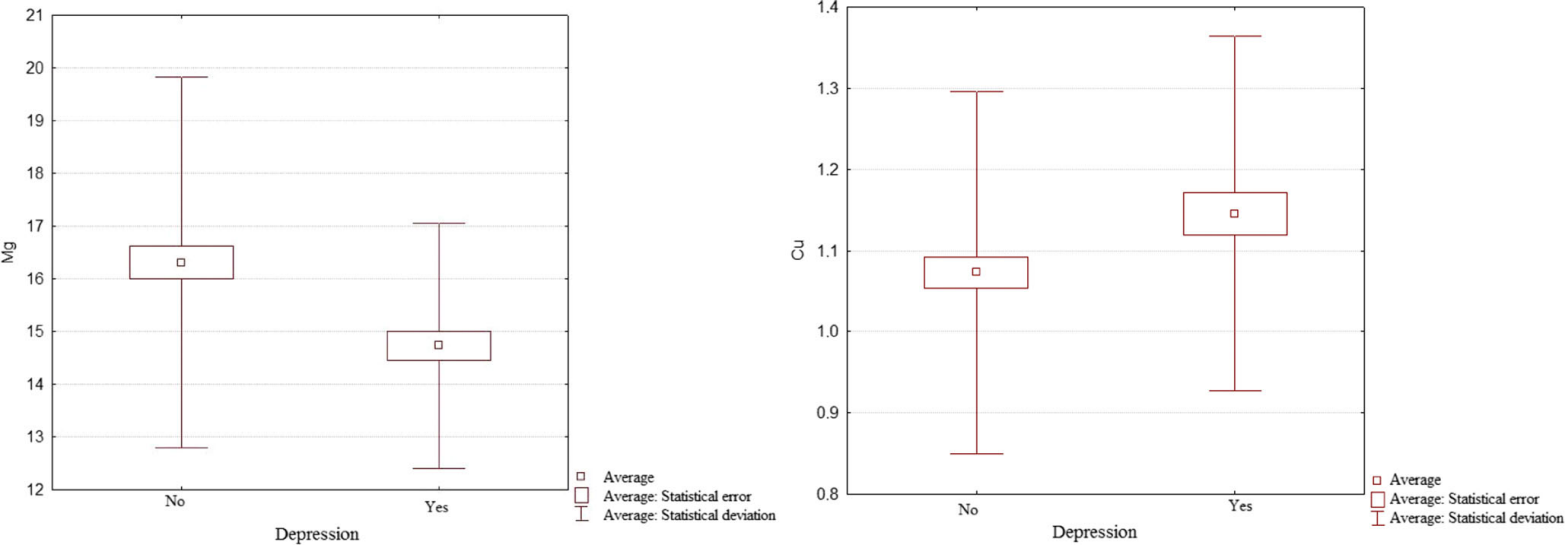
The concentration of Mg and Cu in blood serum of postmenopausal women, and the presence or lack of depression

Data analysis showed statistically significant differences in the concentrations of Mg and Cu. The average concentration of Cu in the entire group of the studied women amounted to 1.10 ± 0.22 mg/l and was within the range accepted as the norm (0.97–1.17 mg/l). The lowest concentration of Cu was observed in women without symptoms of depressiveness (1.07 ± 0.22 mg/l) and the highest in women with severe depressiveness (1.19 ± 0.17 mg/l), and these differences were statistically significant (*p* ≤ 0.05) (Fig. 1). The lowest concentration of Mg was observed in women with symptoms of severe depressiveness (14.28 ± 2.13 mg/l) and the highest in women without symptoms of depressiveness (16.30 ± 3.51 mg/l), and these differences were statistically significant (*p* ≤ 0.05) (Fig. 1). For the average concentration of Mg (15.75 ± 3.23 mg/l) in blood serum of the studied postmenopausal women, there was deficiency in this micronutrient relative to the standard (18.77–24 mg/l).

The lowest concentration of Fe (1.02 ± 0.33 mg) was observed in women who had no symptoms of depressiveness, and in women with mild symptoms of depressiveness (1.07 ± 0.42 mg). The highest average concentration of Fe (1.27 ± 0.55 mg) in the study group was found in women with severe symptoms of depressiveness. The average concentration of this element in the entire group of the studied postmenopausal women amounted to 1.06 ± 0.36 mg/l and was within the range accepted as the norm (0.5–1.7 mg/l). In the case of Zn, the lowest average concentration was found in women with severe depressiveness (0.61 ± 0.07 mg/l), and the highest in women with mild depressiveness (0.74 ± 0.17 mg/l), and no symptoms of depressiveness (0.71 ± 0.40 mg/l). The average concentration of Zn in the entire group of postmenopausal women was 0.70 ± 0.33 mg/l and was lower than the norm for this micronutrient (0.75–1.3 mg/l). The highest concentration of Ca was observed in women without symptoms of depressiveness (73.25 ± 12.04 mg/l) and the lowest in women who showed severe symptoms of depressiveness (67.13 ± 11.65 mg/l). The average concentration of this micronutrient in the entire group of the studied women was 72.59 ± 11.78 mg/l and was lower than the value that is commonly accepted as the norm (81–105 mg/l) (Table 1).

**Table 1:** The average concentration of Mg, Zn, Ca, Cu, and Fe in blood serum of women and the severity of depressiveness

Severity of depressiveness	*n*	$\overline{X}\pm SD$	Min-Max	Me	Q_1−_Q_3_
Mg (mg/l)					
No depression	128	16.30 ± 3.51	8.69–27.17	15.50	13.99–18.33
Mild	39	14.64 ± 2.35	10.50–21.61	14.54	12.62–15.35
Moderate	21	15.10 ± 2.42	10.20–20.20	14.54	13.84–16.56
Heavy	10	14.28 ± 2.13	10.71–17.27	14.29	13.03–16.36
Total	198	15.75 ± 323	8.69–27.17	15.15	13.73–17.57
Zn (mg/l)					
No depression	128	0.71 ± 0.40	0.43–4.82	0.64	0.57–0.74
Mild	39	0.74 ± 0.17	0.46–1.12	0.72	0.61–0.85
Moderate	21	0.63 ± 0.13	0.46–0.98	0.63	0.54–0.68
Heavy	10	0.61 ± 0.07	0.53–0.72	0.60	0.55–0.66
Total	198	0.70 ± 0.33	0.43–4.82	0.65	0.57–0.77
Ca (mg/l)					
No depression	128	73.25 ± 12.04	41.10–109.28	74.03	65.35–81.76
Mild	39	72.52 ± 12.18	52.21–110.59	70.39	64.34–82.72
Moderate	21	71.30 ± 9.19	50.70–87.06	73.12	66.66–75.04
Heavy	10	67.13 ± 11.65	48.38–85.14	70.24	54.64–75.14
Total	198	72.59 ± 11.78	41.10–110.59	73.42	64.74–80.90
Cu (mg/l)					
No depression	128	1.07 ± 0.22	0.46–1.71	1.04	0.92–1.25
Mild	39	1.12 ± 0.22	0.46–1.60	1.13	1.05–1.24
Moderate	21	1.16 ± 0.24	0.78–1.58	1.09	0.99–1.35
Heavy	10	1.19 ± 0.17	0.90–1.50	1.13	1.11–1.30
Total	198	1.10 ± 0.22	0.46–1.71	1.08	0.95–1.26
Fe (mg/l)					
No depression	128	1.02 ± 0.33	0.29–1.78	1.00	0.79–1.29
Mild	39	1.07 ± 0.42	0.34–2.04	1.07	0.76–1.27
Moderate	21	1.13 ± 0.24	0.82–1.81	1.08	1.00–1.20
Heavy	10	1.27 ± 0.55	0.38–2.21	1.10	0.98–1.57
Total	198	1.06 ± 0.36	0.29–2.21	1.05	0.84–1.28

*n* the number of surveyed women, $\overline{X}\pm SD$ average deviation, *Min-Max.* minimum and maximum values, *Me* median, *Q*_1_ lower quartile, *Q*_3_ higher quartile

The analysis of data resulting from the assessment of concentration of micronutrients in postmenopausal women was made taking into account women whose self-assessment showed symptoms of depressiveness (*n* = 70) (mild, medium, and severe), and women who were not diagnosed with such symptoms (*n* = 128). The concentration of Mg was significantly higher in women without symptoms of depressiveness than in women who had experienced these symptoms. Moreover, statistically significant differences (*p* ≤ 0.05) for the concentration of Cu were observed; lower concentration occurred in women without symptoms of depressiveness and higher in women diagnosed with symptoms of depressiveness (Table 2).

**Table 2:** The average concentration of Mg, Zn, Ca, Cu, and Fe in blood serum of postmenopausal women with no symptoms of depressiveness and with symptoms of depressiveness

Micronutrients	No depressiveness *n* = 128	Depressiveness *n* = 70	t/Z	*p*
	$\overline{X}\pm SD$	$\overline{X}\pm SD$		
Mg (mg/l)	16.31 ± 3.51	14.73 ± 2.33	3.37876t	≤0.05
Zn (mg/l)	0.71 ± 0.40	0.69 ± 0.15	0.48195t	>0.05
Ca (mg/l)	73.25 ± 12.04	71.39 ± 11.28	1.286800Z	>0.05
Cu (mg/l)	1.07 ± 0.22	1.14 ± 0.22	−2.42699Z	≤0.05
Fe (mg/l)	1.03 ± 0.33	1.12 ± 0.40	−1.20896Z	>0.05

$\overline{X}\pm SD$ average deviation, *t/Z* Mann-Whitney’s test probability coefficient

The analysis of data showed a statistically significant (*p* < 0.05) negative correlation between the concentration of Mg and depressiveness and a statistically significant (*p* ≤ 0.05) positive correlation between the concentration of Cu and depressiveness (Table 3).

**Table 3:** The analysis of correlation between severity of depressiveness and the concentration of Mg, Zn, Ca, Cu, and Fe in blood serum of the studied women

Severity of depressiveness				
Micronutrients	n	*R*	t (N-2)	*p*
Mg (mg/l)	198	−0.212301	−3.04154	<0.05
Zn (mg/l)	198	−0.017664	−0.24734	>0.05
Ca (mg/l)	198	−0.102752	−1.44618	>0.05
Cu (mg/l)	198	0.179406	2.55310	<0.05
Fe (mg/l)	198	0.102543	1.44321	>0.05

*R* Spearman’s rank correlation coefficient, *t* (*N-2*) statistics of test that verifies the significance of the R correlation coefficient, *p* significance level calculated for R

## Discussion

Analysing the effect of various concentrations of micronutrients on severity of depressiveness and anxiety among the studied women, a negative correlation between the concentration of Mg in blood serum and severity of depressiveness in the surveyed women was demonstrated (*p* < 0.05) and a significant positive correlation between the concentration of Cu and depressiveness in the studied population of female patients (*p* < 0.05). For concentrations of Fe, Ca, and Zn in blood, the occurrence of depressiveness in the surveyed women no significant correlations were shown (*p* > 0.05).

Reports concerning the concentration of Mg in blood serum in the course of depression are not clear. Research carried out by Frizel et al. showed that the concentration of Mg in patients with depression was substantially reduced, and an effective electroconvulsive therapy or tryptophan-based therapy correlate with normalization of the concentration of this element in plasma of the respondents [23]. In research by Zięba et al., it was shown that there was a slight reduction in the concentration of Mg in blood serum of patients with depression compared to the control group. In this case, the severity of depression assessed using Hamilton’s scale did not correlate with the concentration of Mg [24]. In the publication of Widmer et al., higher levels of Mg in blood serum of patients with unipolar depression compared to healthy volunteers were observed, and there was no relation between the concentration of the micronutrient and the mental state of the respondents [25]. Fitzpatrick et al. indicate that Mg deficiency in postmenopausal women can cause or exacerbate depressive symptoms [4]. Research that assesses the level of Mg in blood serum made among 650 people diagnosed with depression in Iraq showed that the mean total serum magnesium was 2.1 ± 0.26 mg/dl. In the study group consisting of both men (30 %) and women (70 %), it was shown that hypomagnesaemia occurred in 13.7 %, hypermagnesaemia in 8.3 %, and the sub-optimal magnesium levels were present in 26.5 %. There was a significant relationship between depression and the level of Mg in blood serum (*p* = 0.02). Hypomagnesaemia influenced the severity of depression in a significant manner, which strongly suggests an important role of Mg in pathogenesis of depression [10].

McNair et al. reported an increased excretion of Mg by kidneys in postmenopausal women. It was found that the use of MHT reduces excretion of Mg in urine [26]. This relationship, however, was not confirmed by Bednarek-Tupikowska. She analysed 120 women, divided into a group using MHT and a group free of the therapy, and did not observe any significant differences in the concentrations of Mg in blood serum between these two groups of women [27].

The available literature on occurrence of depressiveness in postmenopausal women shows that there are significant differences in the frequency and severity of depressive symptoms between menopausal women undergoing the hormone therapy, and those who did not apply it [28]. Janicka et al. studied 60 postmenopausal women who used the hormone therapy, and 30 patients who did not use MHT. To assess symptoms of depression, Beck’s depression scale was used. The overall rate of depression was significantly higher in women who did not use the hormone therapy compared to women who used MHT. In the group of women not using MHT symptoms of depressiveness are more frequently diagnosed, and they are more severe than in the case of patients who use it [29]. The specificity of depressive disorders in the menopausal period is based on similarity of symptoms to those which occur as a result of oestrogen deficiency. In both cases, there is insomnia, mood swings, anxiety, irritability, difficulty concentrating, memory issues, and loss of libido. The clinical picture in the menopausal period includes symptoms of concomitant diseases and a higher level of psychomotor agitation and anxiety [30]. There are attempts to define laboratory markers of depression among which there are hormonal markers (such as the dexamethasone suppression test, thyrotropin or fenfluramine stimulation test), immunological, serum lipids, as well as blood cell markers, amino acids, monoamines, neurodegeneration markers, and some micronutrients.

Among micronutrients, particular attention is paid to the role of Zn, Mg, and Cu as essential modulators of glutamate transmission that are connected with etiopathogenesis of depression [30–32]. Literature indicates that in many research studies carried out among patients diagnosed with depression hypocalcaemia, hypomagnesaemia, and/or lack of Zn and Fe were found [33].

Hansen et al.’s reports signalled that in patients with refractory depression the concentration of Zn in blood serum is significantly reduced [34]. McLoughlin and Hodge presented research which showed that patients diagnosed with depression were characterized by a significant reduction in the concentration of Zn in blood serum, and that the regression of symptoms of depression is accompanied by an increase in the concentration of this micronutrient [35]. Also, other researchers, such as Maes et al., Nowak et al., and Schlegel-Zawadzka et al., provided evidence for a negative correlation between the severity of depression and the concentration of Zn in plasma [36–38]. Siwek et al. studied 30 patients aged 18–55 diagnosed with recurrent depression treated for 12 weeks with imipramine in a daily dose of 100–200 mg and a control group of 25 volunteers. It was shown that during the period of acute symptoms of depression the concentration of Zn was significantly lower than in healthy volunteers. In that research, there was no significant correlation between the concentration of Zn and severity of depression measured using Beck’s depression inventory or Hamilton’s depression scale. In patients who met the criteria of remission, the concentration of Zn in blood serum was statistically significantly higher. The authors concluded that the concentration of Zn in plasma is a marker that indicates presence of the state of depression but does not reflect severity of symptoms of depression [30]. Research conducted by Nowak shows that Zn homeostasis in the human body plays an important role in the mechanism of treatment with antidepressants [14].

Prospective cohort research shows that taking high doses of Zn in a diet is associated with a decrease in incidence of depression. This relation concerned both women and men [39]. In a randomized blinded and placebo-controlled study, it was shown that dietary supplements containing Zn had influence on improvement of mood, reduction of anger, and hostility [40].

In research by Jurczak et al. conducted on a group of 323 postmenopausal women, of which 152 applied MHT and 171 were free of the therapy, it was demonstrated that women using MHT presented high levels of Mg and Zn in blood serum compared to the control group [41].

In our research, we demonstrated a statistically significant (*p* < 0.05) positive correlation between the concentration of Cu in blood and severity of depression in analysed patients. The lower concentration of Cu occurred in women without symptoms of depressiveness (1.07 ± 0.22 mg/l) and higher (1.14 ± 0.22 mg/l) in women diagnosed with depressiveness. Similar results were obtained by Manser et al. [42] and Narang et al. [43]. In both cases, the concentration of Cu was studied among patients diagnosed with a history of depression by comparing them with healthy volunteers. Patients with symptoms of depression presented a statistically significant high concentration of this element compared to the control group. It was also shown that an effective antidepressant therapy results in a significant reduction in the concentration of Cu in blood serum of the respondents [43]. Similar results were also obtained by Schlegel-Zawadzka et al. [44], who studied 19 patients diagnosed with unipolar depression and a group of 16 healthy volunteers. The average concentration of Cu in patients with depression was higher by 21 % than in the control group. In this research, no correlation was obtained between severity of depression measured using Hamilton’s depression scale and the concentration of Cu in blood serum of patients.

Different results were obtained by Maes et al. [45] comparing the concentration of Cu in blood serum of 31 patients diagnosed with major depression, and 15 healthy volunteers. Before starting the treatment of patients diagnosed with depression, the concentration of Cu showed no significant differences. However, a statistically significant decrease in the concentration of Cu among respondents with depression during effective treatment was observed.

## Conclusions

1. The postmenopausal women analysed in this study had decreased levels of Zn, Mg, and Ca compared to the reference values.
2. What is striking is a potential relation between the levels of Mg and Cu and depressiveness. Our results indicate to a higher vulnerability to depression in a group of women with lower levels of Mg and higher levels of Cu.

## Abbreviations

Mg: Magnesium
Zn: Zinc
Ca: Calcium
Cu: Copper
Fe: Iron
FSH: Follicle-stimulating hormone
MHT: Menopausal hormone therapy
